# Palm kernel cake extract exerts hepatoprotective activity in heat-induced oxidative stress in chicken hepatocytes

**DOI:** 10.1186/1472-6882-14-368

**Published:** 2014-10-02

**Authors:** Ehsan Oskoueian, Norhani Abdullah, Zulkifli Idrus, Mahdi Ebrahimi, Yong Meng Goh, Majid Shakeri, Armin Oskoueian

**Affiliations:** Institute of Tropical Agriculture, Universiti Putra Malaysia, Serdang, Selangor 43400 Malaysia; Agricultural Biotechnology Research Institute of Iran (ABRII), East and North-East Branch, P.O.B. 91735/844, Mashhad, Iran; Department of Biochemistry, Faculty of Biotechnology and Biomolecular Sciences, University Putra Malaysia, Serdang, Selangor 43400 Malaysia; Department of Veterinary Preclinical Sciences, Faculty of Veterinary Medicine, Universiti Putra Malaysia, Serdang, Selangor 43400 Malaysia; Ferdowsi University of Mashhad, International Branch, Mashhad, Iran

**Keywords:** Hepatoprotective, Palm kernel cake, Oxidative stress, Chicken hepatocyte, Hsp70, iNOS

## Abstract

**Background:**

Palm kernel cake (PKC), the most abundant by-product of oil palm industry is believed to contain bioactive compounds with hepatoprotective potential. These compounds may serve as hepatoprotective agents which could help the poultry industry to alleviate adverse effects of heat stress on liver function in chickens.

**Methods:**

This study was performed to evaluate the hepatoprotective potential of PKC extract in heat-induced oxidative stress in chicken hepatocytes. The nature of the active metabolites and elucidation of the possible mechanism involved were also investigated.

**Results:**

The PKC extract possessed free radical scavenging activity with values significantly (p < 0.05) lower than silymarin as the reference antioxidant. Heat-induced oxidative stress in chicken hepatocyte impaired the total protein, lipid peroxidation and antioxidant enzymes activity significantly (p < 0.05). Treatment of heat-induced hepatocytes with PKC extract (125 μg/ml) and silymarin as positive control increased these values significantly (p < 0.05). The real time PCR and western blot analyses revealed the significant (p < 0.05) up-regulation of oxidative stress biomarkers including TNF-like, IFN-γ and IL-1β genes; NF-κB, COX-2, iNOS and Hsp70 proteins expression upon heat stress in chicken hepatocytes. The PKC extract and silymarin were able to alleviate the expression of all of these biomarkers in heat-induced chicken hepatocytes. The gas chromatography-mass spectrometry analysis of PKC extract showed the presence of fatty acids, phenolic compounds, sugar derivatives and other organic compounds such as furfural which could be responsible for the observed hepatoprotective activity.

**Conclusion:**

Palm kernel cake extract could be a potential agent to protect hepatocytes function under heat induced oxidative stress.

## Background

Malaysia is one of the major palm oil producers in the world and palm kernel cake (PKC) is the most abundant by-product of this industry [1]. The production of PKC in Malaysia in the year 2013 reached 2,516,664 tons and it is increasing annually [2]. This by-product has been used extensively as a feed ingredient for ruminants, but to a limited extent as poultry feed. Previous studies have demonstrated the wound healing, anti-hyperglycaemic and analgesic activity of different palm species, [3–6] which indicate the presence of bioactive compounds in this plant. Chemical analyses of PKC showed the presence of hemicellulose, cellulose, lignin, proteins, amino acids, oligosaccharides, phenolics and phytosterols [6]. These compounds possess functional groups such as methyl, hydroxyl and carboxyl groups which may offer biological and therapeutic activities [7]. Furthermore, PKC has been shown to contain phenolic antioxidants such as flavonoids, polyphenols and phenolic acids as well as water-soluble vitamins and organic acids [3]. However, information regarding the function of phytochemicals of PKC are rather limited and inconclusive.

Poultry industry is one of the major contributors of food protein in Malaysia. Under the hot and humid environment, the birds are prone to heat stress and liver malfunction which would reduce growth performance and meat quality. This issue remains a major constraint in the large poultry farms in the country. Recent studies have suggested the application of natural bioactive compounds to alleviate the adverse effects of heat stress [8, 9]. Thereby, it might be promising to consider PKC as a source of bioactive phytochemical compounds for therapeutic purposes. The supply of valuable phytochemicals from a reliable and continuous source such as PKC would ensure a sustainable poultry industry.

Hence, this study was conducted to evaluate the hepatoprotective properties of PKC extract in heat-induced oxidative stress in chicken hepatocytes. The phytochemical composition of the extract and possible mechanism involved were also investigated.

## Methods

### Plant material

Palm kernel cake was purchased from Palm Oil Mill Sdn Bhd., Dengkil, Selangor, Malaysia. The PKC sample was dried using a freeze dryer (Labconco, Kansas City, USA). After drying, the PKC was finely ground (mesh 100) using a mechanical grinder. Samples were kept at -20°C for further analyses.

### Microwave-ultrasound assisted extraction

Ten grams of dried fine powder of PKC were placed in a 250 ml of round-bottom flask. One hundred milliliters of 80% aqueous ethanol (v/v) were added. The mixture was heated in a microwave (280 W) for 5 min. Thereafter, the flask was transferred to the ultrasonic bath (Branson Ultrasonic, Danbury, USA) and the frequency and power was adjusted to 20 kHz and 200 W, respectively. A discontinuous process with 30 s ON/OFF cycles to a total time of 5 min was used to extract the PKC. The Whatman No.1 (Whatman, England) filter paper was used to filter the extract and the filtrate was concentrated to dryness using a Rotary Evaporator (Buchii, Switzerland). The stock concentration of 100 mg/ml using dimetyl sulfoxide was prepared and kept at -80°C for further analyses [10].

### Radicals scavenging activity

#### 2, 2-diphenyl-1-picrylhydrazyl scavenging activity

The 2, 2-diphenyl-1-picrylhydrazyl (DPPH) free radical was used to determine the scavenging activity of the extract [11]. Briefly, 1 ml of PKC extract at different concentrations were prepared and mixed with 3 ml of 0.1 mM solution of DPPH methanolic solution. The mixture was incubated at room temperature for 30 min in dark condition. The absorbance of the samples was read at 517 nm using a spectrophotometer (Molecular Devices, Sunnyvale, CA). Silymarin (Sigma, S0292, MO, USA) was used as the reference antioxidant in this test.

### ABTS scavenging activity

The solution of 7 mM 2,2 azino bis (3-ethylbenzothiazoline-6sulfonic acid) (ABTS) was prepared using water. Then, the potassium persulfate was added to reach the final concentration of 2.45 mM. The mixture was incubated at room temperature in the dark for 16 h. The absorbance was adjusted to 7 ± 0.01 at 734 nm using ultra-pure water. A 100 μl of PKC extract was added to 900 μl of this diluted solution and incubated for 2 min in dark condition. Then, the absorbance was read at 734 nm using a spectrophotometer (Molecular Devices, Sunnyvale, CA). The percentage reduction in the absorbance indicated the extent of decolorization [12]. The silymarin was used as a reference antioxidant.

### Nitric oxide scavenging activity

The nitric oxide (NO) scavenging activity of extract was evaluated as described by Tsai et al. [13]. A series of concentrations (0 - 400 μg/ml) of PKC extract was prepared and 60 μl of PKC extract in different concentration was mixed with 10 mM sodium nitoprusside in phosphate buffered saline (PBS). The mixture was incubated at room temperature for 150 min. Finally, 60 μl of Griess reagent (1 g/L sulfanilamide, 25 g/L phosphoric acid, and 0.1 g/L N-1-naphthylethylenediamine) were added to determine the remaining free NO in the reacting solution. The absorbance was determined at 550 nm using a spectrophotometer (Molecular Devices, Sunnyvale, CA). The silymarin was used as a reference antioxidant.

### Isolation and culture of primary chicken hepatocytes

In order to isolate the hepatocytes, 2-step collagenase method was applied [14]. The 5-wk-old male chickens were treated by natrium thiopenthal (40 mg/kg) and heparin (1,600 U/kg) through intra-peritoneal injection. After full anaesthesia, the abdominal cavity was opened to have easy access to the liver. The livers were prefused with different types of buffers as described by Wang et al. [14]. After the prefusion steps, the livers were excised and digested using 0.4 mg/ml of collagenase type IV for 20 min at 37°C. The digestion was stopped by adding a William’s E medium (Gibco, Grand Island, NY) which was supplemented with 5% chicken serum and 2 mg/ml bovine serum albumin (BSA). Cells were passed through 100, 60, 30 μm filters. The cells were incubated with red blood cells lysis buffer and washed using William’s E medium containing chicken serum to eliminate the red blood cells. The viability of isolate cells was evaluated using trypan blue test and cells were cultured in William’s E medium supplemented with 100 U/ml of penicillin-streptomycin, 10 μg/ml insulin and 5% chicken serum. The cells were incubated at 37°C with 5% CO_2_ in a humidified incubator. The approval of Animal Use and Care Committee (ACUC), Faculty of Medicine and Health Sciences, University Putra Malaysia for this procedure was obtained.

### Cytotoxicity assay

The cytotoxicity effect of PKC extract on chicken hepatocytes was determined using MTT assay [15]. The cells were grown in 96-well plates with the density of 5 × 10^3^ cells/100 μl of medium. Serial concentrations of PKC extract were prepared from 50 to 1000 μg/ml. The cells were treated with the extract and incubated for 24 h. Finally, the viability of the cells was determined by using 3-(4,5-Dimethylthiazol-2-yl)-2,5-Diphenyltetrazolium Bromide (MTT). The silymarin was used as a positive control.

### Induction of acute heat shock

The isolated chicken hepatocytes were cultured as mentioned above. When the confluency reached to 90%, the cells were trypsinized and harvested. Cells were cultured in T25 plates at the density of 5 × 10^3^ cells/ 100 μl of medium. Then cells were incubated at 37°C with a humidified atmosphere of 5% CO_2_ for 12 hours. The media were removed and replaced with fresh medium containing PKC extract at concentration of 125 μg/ml. The negative control was incubated at 37°C in a humidified atmosphere of 5% CO_2_ while the heat-induced cells were incubated at 40°C in a humidified atmosphere of 5% CO_2_ for 180 min [15]. The positive control was heat-induced cells treated with silymarin.

### Antioxidant enzyme assay

Upon treatments, cells were rinsed using ice-cold 0.1 M phosphate buffered saline (PBS) pH 7.4. Then, 5 ml PBS were added and cells were scraped and transferred into 15 ml centrifuge tube. Cells were centrifuged at 250 × g for 15 min at 4°C and the supernatant was removed and cells were suspended in PBS and incubated in cell-lysis solution for 30s, centrifuged at 2800 × g for 10 min at 4°C. The supernatant was collected and the antioxidant enzymes activity was determined. The kits used to determine the superoxide dismutase activity (SOD), catalase activity (CAT) and glutathione reductase activity (GR) were from Nanjing Jiancheng Bioengineering Institute (Nanjing, China) and the assay methods were according to the instructions provided by the kits [16]. All the results were expressed as enzyme activity/g protein (U/g protein) of the cells. The silymarin was used as a positive control.

### Lipid peroxidation assay

The malondialdehyde (MDA) as end product of lipid peroxidation was determined in the chicken hepatocytes using thiobarbituric acid reactive substance (TBARS) assay [17]. The cultured cells upon treatment were rinsed with PBS buffer for three times and scraped. The cells were transferred into 4 ml of potassium chloride (1.15%), kept on ice and homogenized using Ultra-Turrax homogenizer (Wilmington, NC, USA) at 20,000 rpm for 30 s. Then, a 200 μl of homogenized sample, 300 μl distilled water, 35 μl BHT, 165 μl sodium dodecyl sulphate (SDS) and 2 ml TBA were added in the screw cap test tube. The mixture was heated for 60 min at 90°C. The solution was cooled immediately and 3 ml of n-butanol was added. Tubes were centrifuged at 2800 × g for 10 min. The absorbance of n-butanol fraction was read at 532 nm by spectrophotometer (Molecular Devices, Sunnyvale, CA). The standard curve was constructed using different concentration of 1,1,3,3-tetraethoxypropane (2.5-50 μM). The silymarin was used as a positive control.

### Molecular biomarkers of oxidative stress

#### Gene expression analyses

Cells were cultured and treated as mentioned above. The media were removed and cells were washed twice with ice-cold PBS and scraped. The RNasey mini kit (Qiagen, Valencia, CA, USA) was used to extract the RNA. The quality of the total RNA (concentration, integrity, size distribution) was determined. The extracted RNA was then converted to cDNA with reverse transcriptase PCR (RT-PCR) using Maxime RT Permix kit (iNtRON Biotechnology, Sungnam, Korea). The expression of tumor necrosis factor-like ligand 1A (TNF-like), interleukin-1 beta (IL-1β), interferon gamma (IFN-γ) and glyceraldehyde 3-phosphate dehydrogenase (GAPDH) and β-actin genes (Table 1) were analysed using iQ SYBR Green Supermix (Bio-Rad) on a Real-time PCR thermocycler (Bio-Rad, CA, USA) [18]. In chickens, the TNF-α has not been identified, thus, TNF-like ligand 1A (TNF-like) was analysed which produced effects similar to TNF-α. The optimum condition for the amplification of all genes were as follow: 94°C for 5 min (1X), then 94°C for 20 s, then 60°C for 20 s and 72°C for 25 s (35X). The Vandesompele et al. [19] method was used to normalize the genes to the GAPDH and β-actin expression. The real time PCR results were analysed using CFX manager software version 2 (Bio-Rad Laboratories).

**Table 1:** **The primer characteristics used for the gene expression analysis**

Genes		Sequences (5′ to 3′)	References
TNF-like^1^	F	tgctgttctatgaccgcc	[20]
	R	ctttcagagcatcaacgca	
IL-1β^2^	F	atg gcgttcgttcccgacctggacgtgctg	[21]
	R	acttagcttgtaggtggcgatgttgacctg	
IFN-γ^3^	F	gctgacggtggacctattatt	[21]
	R	tggattctcaagtcgttcatcg	
GAPDH^4^	F	tgaaagtcggagtcaacggatt	[22]
	R	ccacttggactttgccagaga	
β-Actin	F	caacacagtgctgtctggtgg	[20]
	R	atcgtactcctgcttgctgat	

^1^In chickens, TNF-α has not been identified. but TNF-like ligand 1A (TNF-like) produced effects similar to TNF-α.

^2^Interleukin-1 beta.

^3^Interferon gamma.

^4^Glyceraldehyde 3-phosphate dehydrogenase.

### Western blot analysis

The expression of nuclear factor kappa-light-chain-enhancer of activated B cells (NF-κB), nitric oxide synthase (iNOS), cyclooxygenase-2 (COX-2), 70 kilodalton heat shock protein (Hsp70) and β-actin proteins were determined by Western blot analysis. The chicken hepatocytes were cultured and treated as mentioned above. Cells were trypsinized and washed with ice-cold PBS. The 100 μl of lysis buffer (0.5% Triton X-100, 2 mM EDTA in 20 mM Tris-HCL pH 7.5) containing 10 μl/ml of protease inhibitor (ProteoBlock Protease Inhibitor Cocktail, Fermentas, MD, USA) was used to lyse the cells at 4°C. The cells were then sonicated for 15 s using a UP 100H sonicator (Hielscher, Germany) and incubated on ice for 20 min. The cells lysate were centrifuged at 14,000 × g for 30 min and the supernatant was collected. The Protein Assay kit (Bio-Rad, CA, USA) was used to measure the concentration of protein in the supernatant. The 20 μg of protein was denatured by incubation at 95°C for 5 min and subjected to electrophoresis using Tris-glycine polyacrylamide gel. Proteins were transferred to a PVDF membrane using Hoefer Semi-Dry Transfer Unit (Hoefer Instruments, CA, USA). Upon protein transfer, the Odyssey Blocking Buffer (LI-COR, Lincoln, NE, USA) was used to wash the membrane. The membrane was then incubated overnight with a 1:1000 dilution of NF-κB (Biorbyt orb11124), iNOS (Biorbyt orb13614, COX-2 (Santa Cruz SC7951), Hsp70 (Biorbyt orb10848) and β-actin (Biorbyt orb40714) primary antibodies. The membrane was washed with 0.05% PBST (phosphate buffer saline and Tween 20) three times for 5 min. For detection with the Odyssey imaging system, a 1:10000 dilution of the IRDye 800 CW Goat Anti-Rabbit Secondary Antibody or IRDye 680 Goat Anti-Mouse Secondary Antibody was used. The solution of 0.05% PBST was used to wash the membrane for 5 min and repeated three times. The membrane was dried and visualized using the Odyssey Infrared Imaging System (LI-COR, Lincoln, NE, USA) and Odyssey software was used to determine the intensity of the protein bands [23].

### Metabolites profiling by gas chromatography-mass spectrometry (GC-MS)

The chemical composition of PKC extract was determined using GC-MS QP2010 Plus (Shimadzu Co., Kyoto, Japan) [24]. The GC was equipped with BPX-5 SGE ultra-low-bleed 5% phenyl polydimethylsiloxane capillary column (30 m × 0.25 mm i.d. × 0.25 μm film thickness). Helium was used as the carrier gas at the flow rate of 1 ml/min and the analysis was done splitless with a purge time of 1 min. Initially, the temperature of the column was maintained at 50°C for 3 min, followed by 5°C/min to 80°C and then at 10°C/min to 350°C. The temperature of the inlet and detector was 250°C and 340°C, respectively. The peaks were identified according to computer matching of the mass spectra with the National Institute of Standards and Technology (NIST 08 and NIST 08 s) library and also comparing with the published data.

### Statistical analysis

The GLM procedure of SAS [25] was used to analyse the data in a complete randomized design. Differences in Duncan were considered significant at p < 0.05. The results obtained in Western blot were analysed by GraphPad Prism 5 software (GraphPad Software Inc., San Diego, CA, USA). All measurements were conducted in triplicate.

## Results and discussion

### Radical scavenging activity

The free radical scavenging activity of the PKC extract is presented in Table 2. The PKC extract inhibited 50% of the DPPH, ABTS and NO free radicals at the concentrations of 65.3, 76.2 and 107.4 μg/ml. Silymarin, which is a well-known hepatoprotective agent, [26] was used as reference antioxidant. The IC_50_ concentration of this compound to inhibit the DPPH, ABTS and NO free radicals were 43.5, 56.7 and 61.6 μg/ml, respectively (Table 2). Both PKC extract and silymarin indicated anti radical activity. However, the activity of PKC extract was significantly (p < 0.05) lower than that of silymarin. According to the results in Table 2 and based on the antioxidant potential categorised by Pasias et al. [27] and Tsai et al. [13] the PKC extract and silymarin is considered as good DPPH, ABTS and NO scavengers. The appreciable antioxidant activity may offer resistance toward oxidative stress by inhibiting the free radicals as several *in vivo* studies, [17, 28, 29] indicated that plant extract with noteworthy radical scavenging activity could decrease the inflammatory responses in the rat liver challenged by oxidative stress.

**Table 2:** **Free radical scavenging activity of PKC extract and silymarin**

Scavenging activity	IC _50_ (μg/ml)		S.E.M
	PKC extract	Silymarin	
DPPH^a^	65.3^a^	43.5^b^	6.83
ABTS^b^	76.2^a^	56.7^b^	5.25
NO^c^	107.4^a^	61.6^b^	7.43

^a^2, 2-diphenyl-1-picrylhydrazyl (DPPH).

^b^2,2 azino bis (3-ethylbenzothiazoline-6sulfonic acid).

^c^Nitric oxide.

Another recent report by Ng et al. [30] validated the presence of peptides in the PKC with antiradical activity. In addition, the phenolic compounds extracted from the leaf of palm tree have been shown to inhibit the free radicals similar to that of green tea extract [3].

### Cytotoxicity assay

**Figure 1:**
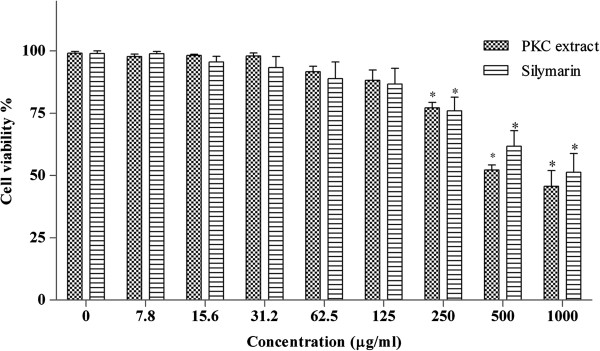
**The cytotoxic effects of PKC extract and silymarin upon 24 h incubation with chicken primary hepatocytes determined by MTT assay.** Values are presented as means ± S.E.M (n = 3).

The results of the cytotoxic effects of PKC extract and silymarin as a positive control on chicken primary hepatocytes are presented in Figure 1. The cell culture model was applied to determine the hepatotoxicity of the PKC extract since the isolated chicken hepatocytes maintained the majority of specialized function like normal liver.

The concentrations of 0-1000 μg/ml for the period of 24 h were tested and the results showed significant (p < 0.05) decrease in the hepatocyte viability at the concentration of 250 μg/ml and above by both PKC extract and silymarin. The concentrations of 125 μg/ml and below of both compounds revealed no toxicity, thus, the concentration of 125 μg/ml was chosen as nontoxic concentration to treat the cells. These results were in agreement with the results reported by Tan et al. [3] who reported the cytotoxic effects of the palm phenolic extract against human and mouse skin melanoma cancer cells. This extract has also been shown to induce the program cells death in the breast and kidney cancer cell lines [31].

### Antioxidant enzymes and lipid peroxidation

The result of total protein, lipid peroxidation and antioxidant enzymes activity in primary chicken hepatocytes is presented in Table 3. The total protein value in untreated cells was 0.5 mg/ml. Induction of heat stress significantly (p < 0.05) increased the value to 0.8 mg/ml. The untreated cells showed the lipid peroxidation value of 1.7 nM MDA/ mg protein. Induction of heat stress significantly (p < 0.05) increased this value to 6.8 nM MDA/mg protein. The activity levels of SOD, CAT and GR in untreated cells were 13.4, 8.9 and 0.7 U/mg protein, respectively. Heat stress in hepatocytes suppressed the activity of these enzymes significantly (p < 0.05) to 4.6, 3.2 and 0.2 U/mg protein respectively.

**Table 3:** **Total protein, lipid peroxidation and antioxidant enzymes activity of chicken hepatocytes under oxidative stress induced by high temperature**

	Treatments				
Items	I	II	III	IV	SEM
Total protein (mg/ml)	0.5^b^	0.8^a^	0.6^b^	0.6^b^	0.03
Lipid peroxidation (nM MDA/mg protein)	1.7^c^	6.8^a^	2.9^b^	2.6^b^	0.38
SOD activity (U/mg protein)	13.4^a^	4.6^c^	8.7^b^	9.3^b^	1.23
CAT activity (U/mg protein)	8.9^a^	3.2^d^	5.8^c^	6.7^bc^	1.16
GR activity (U/mg protein)	0.7^a^	0.2^d^	0.4^c^	0.5^bc^	0.04

I: Untreated Cells.

II: Heat-induced cells.

III: Heat-induced cells + PKC extract (125 μg /ml).

IV: Heat-induced cells + silymarin (125 μg /ml).

MDA: Malondialdehyde as lipid peroxidation biomarker.

SOD: Superoxide dismutase.

CAT: Catalase.

GR: Glutathione reductase.

Means (n = 3) with different superscripts within row are significantly different (P < 0.05).

The SOD, CAT and GR systems work in synergy with free radical scavengers to protect the cell against deleterious effects of reactive oxygen species (ROS). For instance, the SOD enzyme converts the superoxide radicals into H_2_O_2_ and thereafter the activation of CAT and glutathione peroxidase (GPx) enzymes degrade H_2_O_2_ into water. These results imply that, heat stress increased radical formation and interrupted the antioxidant defense mechanism, which resulted in the occurrence of oxidative stress that led to hepatocytes malfunction. The plant extracts with antioxidant activity improve the function of antioxidant enzymes, inhibit lipid peroxidation and enhance detoxification system in various animal models [26, 28, 29]. Similarly, Ramnath et al. [32] observed an increase in lipid peroxidation and reduction in antioxidant enzymes activity in the serum and liver of chickens induced by heat stress. As shown in Table 3, treatment of cells with 125 μg/ml of PKC extract and silymarin showed significant (p < 0.05) restoration of the altered biochemical parameters observed in the untreated heat-induced cells. These finding indicated that PKC extract had protective effects on chicken hepatocytes probably by activating antioxidant mechanism in the cells and inhibiting the chain reaction of lipid peroxidation. Similar observation was made by Das et al. [33] who reported the increase in lipid peroxidation, SOD and catalase enzymes depletion in heat-induced oxidative stress in the rat liver. These parameters were appreciably normalized by treatment of rats with polyphenol compound called resveratrol. Likewise, the curry leaf extract and plant alkaloid called berberine were able to improve the lipid peroxidation, total cellular protein, antioxidant enzymes activity in human and mice liver cells under oxidative stress induced by ethanol and CCl_4_, respectively [28, 34].

### Analyses of molecular biomarkers of oxidative stress

**Figure 2:**
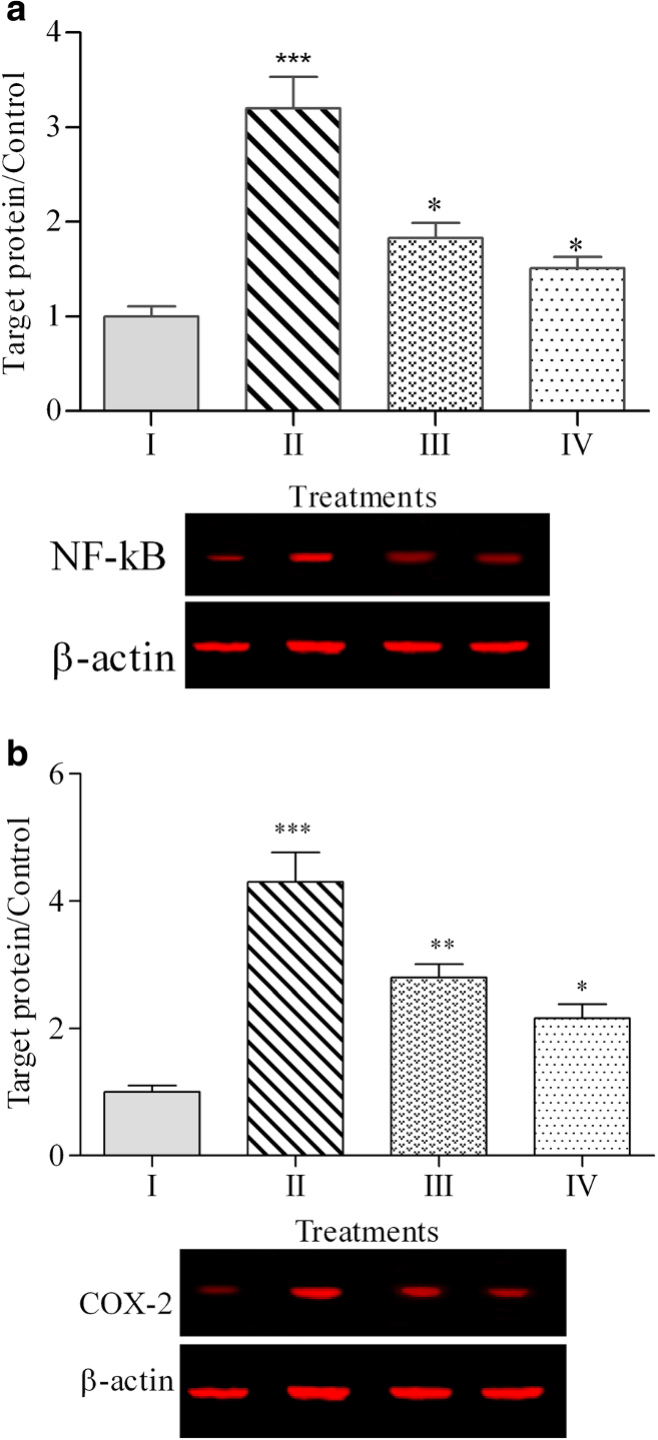
**Expression of NF-κB (a) and COX-2 (b) proteins in chicken hepatocytes.** (I: Untreated cells, II: Heat-induced cells, III: Heat-induced cells + PKC extract, IV: Heat-induced cells + silymarin). Equal amounts of total cellular protein from different treatments were subjected to Western blot analyses for NF-κB, COX-2 and β-actin proteins. The graph represents the mean ± standard error from three independent experiments, *** p < 0.001, ** p < 0.01 and * p < 0.05 indicate significant difference compared to the untreated cells.

**Figure 3:**
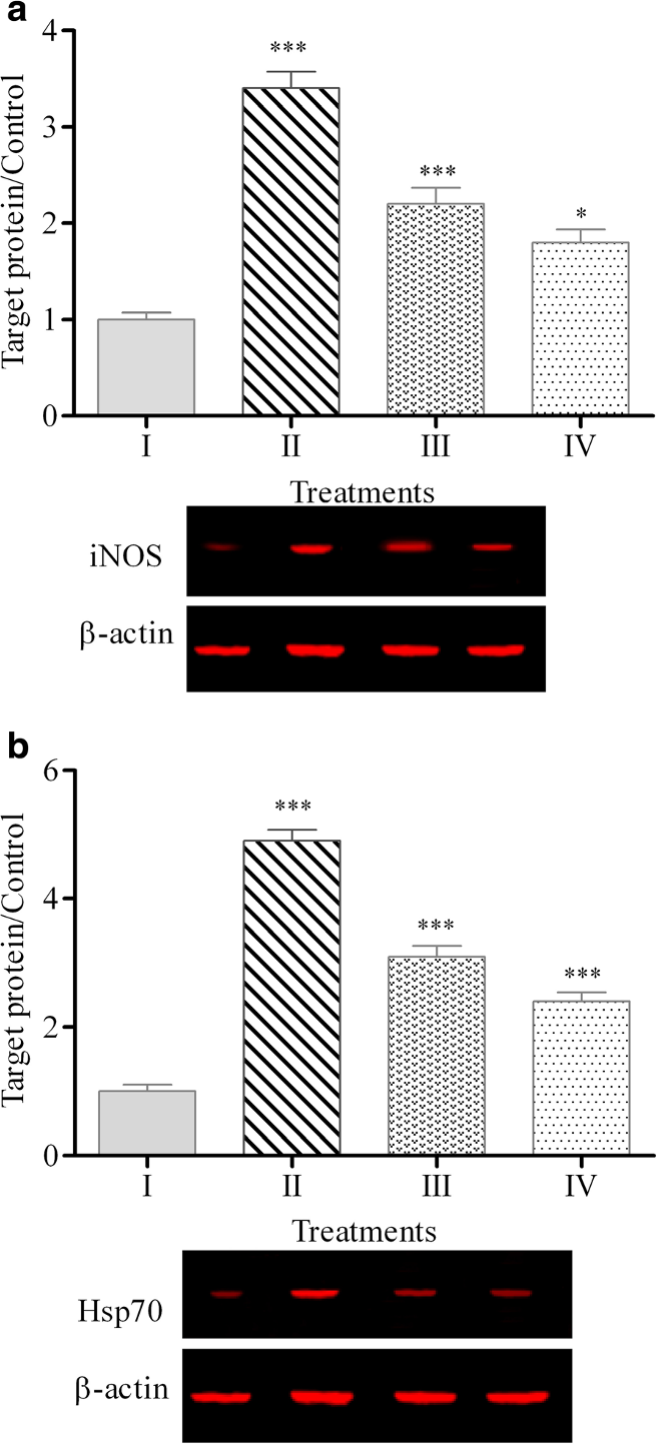
**Expression of iNOS (a) and Hsp70 (b) proteins in chicken hepatocytes.** (I: Untreated cells, II: Heat-induced cells, III: Heat-induced cells + PKC extract, IV: Heat-induced cells + silymarin). Equal amounts of total cellular protein from different treatments were subjected to Western blot analyses for iNOS, Hsp70 and β-actin proteins. The graph represents the mean ± standard error from three independent experiments, *** p < 0.001, ** p < 0.01 and * p < 0.05 indicate significant difference compared to the untreated cells.

The expression of genes in chicken hepatocytes upon various treatments is presented in Table 4. The heat induction significantly (p < 0.05) up-regulated the expression of TNF-like, INF-γ and IL-1β genes with values 6.5, 4.4 and 5.3 folds, respectively. Treatment of heat-induced cells with PKC extract decreased the expression levels of these genes to 3.1, 2.4 and 2.8 folds. Likewise, silymarin also alleviated the expression levels of these genes to 2.7, 2.3 and 2.5 folds, respectively.Figures 2 and 3 illustrate the expression levels of proteins in chicken hepatocytes. The expression levels of NF-κB, COX-2, iNOS and Hsp70 proteins in untreated hepatocytes were 1 ± 0.11, 1 ± 0.14, 1 ± 0.07 and 1 ± 0.12 folds, respectively. These values were significantly (p < 0.05) up-regulated in response to heat stress in chicken hepatocytes to 3.2 ± 0.33, 4.3 ± 0.47, 3.4 ± 0.17 and 4.9 ± 0.17 folds, respectively. As shown in Figures 2 and 3, both PKC extract and silymarin ameliorated the expression of stress proteins as biomarkers of oxidative stress. The PKC extract was able to alleviate the expression of NF-κB, COX-2, iNOS and Hsp70 proteins in heat-induced cells to 1.8 ± 0.16, 2.8 ± 0.21, 2.2 ± 0.16 and 3.1 ± 0.16 folds, respectively. The expression of NF-κB, COX-2, iNOS and Hsp70 proteins for the hepatocytes treated by silymarin were 1.51 ± 0.12, 2.16 ± 0.22, 1.8 ± 0.13 and 2.4 ± 0.14 folds, respectively.

**Table 4:** **Fold-changes in the expression levels of cells upon different treatments**

Analysed Genes	I	II	III	IV	S.E.M
TNF-like	1^c^	6.5^a^	3.1^b^	2.7^b^	0.23
IFN-γ	1^c^	4.4^a^	2.4^b^	2.3^b^	0.12
IL-1β	1^c^	5.3^a^	2.8^b^	2.5^b^	0.16

I: Untreated cells.

II: Heat-induced cells.

III: Heat-induced cells + PKC extract (125 μg /ml).

IV: Heat-induced cells + Silymarin (125 μg /ml).

The expression level of each studied genes was normalized to β-actin and GAPDH.

Means (n = 3) with different superscripts within row are significantly different (P < 0.05).

**Figure 4:**
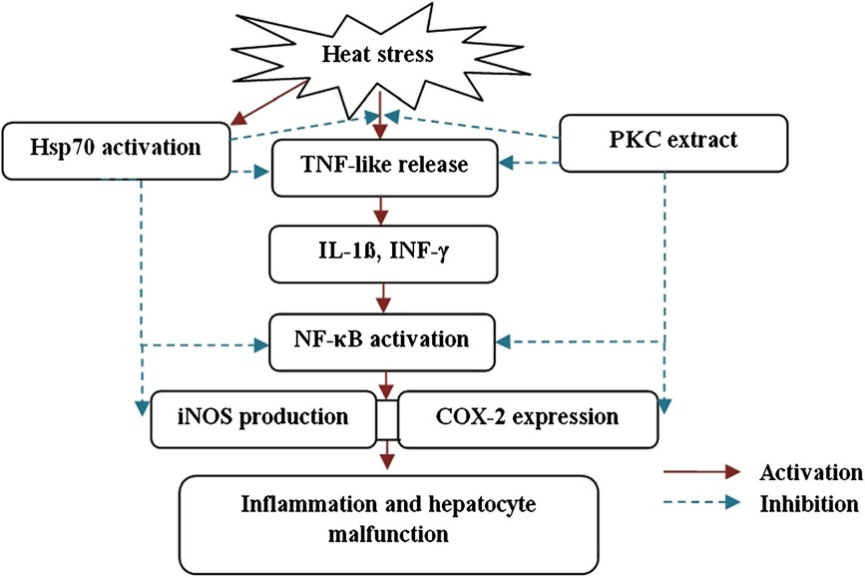
**This scheme indicates the probable mechanisms of hepatoprotective activity of palm kernel cake extract.** The heat stress triggers the TNF-like release in chicken hepatocyte which then activates the proinflammatory cytokines (IL-1β and INF-γ). As a result, NF-κB is activated and it is followed by increase in the expression of COX-2 and iNOS production. The consequence is the inflammation and hepatocyte malfunction. The palm kernel cake exerts the hepatoprotective activity through inhibition of ROS and NO• radicals and inhibiting inflammatory pathways downstream of cytokine release (NF-κB, iNOS and COX-2) and also by decreasing proinflammatory factors TNF-like, IL1-β and INF-γ. The heat stress increases the Hsp70 expression. This over expression alleviates the deleterious effects of heat stress through inhibition of ROS and regulation of TNF-like, NF-κB, iNOS and COX-2 expression.

In this study, heat stress up-regulated the TNF-like gene, causing the up-regulation of IL1-β and INF-γ (Figure 4). It seems that, the elevation in the expression of these genes may end up to the up-regulation of NF-κB protein. The increase in production of NF-κB aggravated the expression of iNOS and COX-2. The increase in production of these proteins induced the production of NO and inflammation response in chicken hepatocyte (Figure 4). It is expected that the more TNF-like production, the higher the intensity of oxidative stress and inflammatory responses. These findings were in accordance with Domitrovic et al. [34] and Deng et al. [28] who suggested the over-expression of iNOS and COX-2 through activation of TNF-α, IL-1β and INF-γ proteins in the rat liver under oxidative stress.

The PKC extract and silymarin exerted hepatoprotective activity by blocking inflammatory pathways downstream of cytokine release (NF-κB, iNOS and COX-2) and also by decreasing proinflammatory factors TNF-like, IL1-β and INF-γ (Figure 4). The reduction in the expression level of iNOS and COX-2 upon treatment by PKC extract is attributed to the regulatory activity and expression of NF-κB. In several experimental models, it has been demonstrated that some plant bioactive compounds could suppress the activation of NF-κB and downstream proinflammatory mediators such as iNOS [26, 28, 29, 34].

The Hsp70 is considered as a cellular defense mechanism against ROS and deleterious effects of oxidative stress. It enhances stress tolerance and thereby increases the cells’ survival. In the current study, the expression of Hsp70 was increased by heat stress. This observation was supported by Yan et al. [35] who reported the increase in the expression of Hsp70 as primary thermo resistance response of the cells to heat stress. The up-regulation of Hsp70 probably contributed to the inhibition of TNF-like, NF-κB, iNOS and COX-2 production (Figure 4). In line with the suggested mechanism, there have been several reports which indicated the capability of Hsp70 to improve the cellular antioxidant enzyme [36], inhibit TNF-α [37], regulate NF-κB [38], attenuate inflammatory responses and decrease iNOS production [39].

### GC-MS analyses of PKC extract metabolites

The principle metabolites in the PKC extract identified by GC-MS are presented in Table 5. The analysis revealed the presence of fatty acids (lauric acid, myristic acid, palmitic acid, caprylic acid), sugar derivatives (β-d-talopyranose, isosorbide), phenolic compounds (4-Hydroxybenzoic acid, 4H-Pyran-4-one) and other organic compound such as furfural. Overall, the PKC extract was composed of 43.9% fatty acids, 20.1% sugar derivatives, 18.2% phenolic compounds and 6.6% furfural. The remaining 11.2% of the extract contained various miscellaneous organic compounds with negligible concentrations.

**Table 5:** **The major metabolites detected in the PKC extract**

Compounds	Content% (w/w)
Lauric acid	18.4
Myristic acid	13.5
Hydroxybenzoic acid	12.3
β-d-talopyranose	11.6
Isosorbide	8.5
Furfural	6.6
Palmitic acid	6.6
4H-Pyran-4-one	5.9
Caprylic acid	5.4
Others	11.2

Currently, little information is available on the active metabolites of PKC, thus comparison was made to metabolites reported in other palm plant materials in previous studies. Tan et al. [3] reported the presence of hydroxybenzoic acid, cinnamic acid, ferulic acid and coumaric acid, and the flavonoid rutin hydrate in the palm leaf. Other bioactive compounds such as vitamin A, thiamine and riboflavin were also reported in the oil of raphia palm [40]. Further, the oil was also reported to contain β-sitosterol, stigmasterol, campesterol and saponins [6]. The hepatoprotective activity of the PKC could be attributed to the presence of fatty acids, sugars and most likely phenolic compounds. In line with these results, Makni et al. [41] and Maheswari et al. [42] demonstrated the hepatoprotective activity of flax, pumpkin and grape seed oils which contained fatty acids and phenolic compounds. The antiradical activity and modulation of the oxidative biomarkers at the transcriptional level were reported to be the mechanism of hepatoprotective action [26].

The results demonstrated that PKC extract showed protective activity against heat stress in the chicken hepatocytes. The involved mechanism was clarified by measuring relevant parameters such as antioxidant activity, cellular antioxidant enzymes, lipid peroxidation, and molecular biomarkers of oxidative stress. In this study, PKC extract performed similar to that of silymarin. Silymarin is a flavonolignan present in the seeds of *Silybum marianum* which enhances the antioxidant property and inhibits inflammatory responses in liver cells [26]. The production of silymarin has been limited as the seed of *Silybum marianum* is the only source. Therefore, in lieu of the limited commercial production of silymarin, PKC, which is abundantly available, should be seriously considered as a source of hepatoprotective extractable metabolites.

## Conclusions

The results of this study suggested that PKC extract was able to protect the chicken hepatocytes against cellular oxidative damage. The possible mechanisms of the hepatoprotective effects, by which the PKC extract protects hepatocyte malfunction, include enhancing the antiradical capability and the intracellular antioxidant enzymes and by lowering lipid peroxidation. The PKC extract also showed the ability to modulate the expression of molecular biomarkers involved in heat-induced oxidative stress in chicken hepatocytes.

## Abbreviations

PKC: Palm kernel cake
DPPH: 2, 2-diphenyl-1-picrylhydrazyl
ABTS: 2,2 azino bis (3-ethylbenzothiazoline-6sulfonic acid
NO: Nitric oxide
PBS: Phosphate buffered saline
BSA: Bovine serum albumin
ACUC: Animal use and case committee
MTT: 3-(4,5-dimethylthiazol-2-yl)-2,5-diphenyltetrazolium bromide
SOD: Superoxide dismutase activity
CAT: Catalase activity
GR: Glutathione reductase activity
MDA: Malondialdehyde
TBARS: Thiobarbituric acid reactive substance
SDS: Sodium dodecyl sulphate
TNF-like: Tumor necrosis factor-like ligand 1A
IL-1β: Interleukin-1 beta
IFN-γ: Interferon gamma
GAPDH: Glyceraldehyde 3-phosphate dehydrogenase
NF-κB: Nuclear factor kappa-light-chain-enhancer of activated B cells
iNOS: Inducible nitric oxide synthase
COX-2: Cyclooxygenase-2
Hsp70: 70 kilodalton heat shock protein
GC-MS: Gas chromatography-mass spectrometry
ROS: Reactive oxygen species
GPx: Glutathione peroxidase.

## References

[1] Cervero JM, Skovgaard PA, Felby C, Sorensen HR, Jorgensen H (2010). Enzymatic hydrolysis and fermentation of palm kernel press cake for production of bioethanol. Enzyme Microb Tech.

[2] *Production of palm kernel cake for the year of 2013*. [http://bepi.mpob.gov.my]

[3] Tan YA, Sambanthamurthi R, Sundram K, Wahid MB (2007). Valorisation of palm by-products as functional components. Eur J Lipid Sci Tech.

[4] Zarei M, Ebrahimpour A, Abdul-Hamid A, Anwar F, Saari N (2012). Production of defatted palm kernel cake protein hydrolysate as a valuable source of natural antioxidants. IJMS.

[5] Hoek-van Den Hil EF, Keijer J, Bunschoten A, Vervoort JJM, Stankova B, Bekkenkamp M, Herreman L, Venema D, Hollman PCH, Tvrzicka E, Rietjens IMCM, Schothorst EMV (2013). Quercetin induces hepatic lipid omega-oxidation and lowers serum lipid levels in mice. PLoS One.

[6] Ofori-Boateng C, Lee K (2013). Sustainable utilization of oil palm wastes for bioactive phytochemicals for the benefit of the oil palm and nutraceutical industries. Phytochem Rev.

[7] Acamovic T, Brooker JD (2005). Biochemistry of plant secondary metabolites and their effects in animals. P Nutr Soc.

[8] Rhoads RP, Baumgard LH, Suagee JK, Sanders SR (2013). Nutritional interventions to alleviate the negative consequences of heat stress. Adv Nutr.

[9] Sahin K, Orhan C, Smith M, Sahin N (2013). Molecular targets of dietary phytochemicals for the alleviation of heat stress in poultry. World Poultry Sci J.

[10] Lianfu Z, Zelong L (2008). Optimization and comparison of ultrasound/microwave assisted extraction (UMAE) and ultrasonic assisted extraction (UAE) of lycopene from tomatoes. Ultrason Sonochem.

[11] Gulcin I, Elmastas M, Aboul-Enein HY (2012). Antioxidant activity of clove oil – A powerful antioxidant source. Arab J Chem.

[12] Kim H, Moon JY, Kim H, Lee DS, Cho M, Choi HK, Kim YS, Mosaddik A, Cho SK (2010). Antioxidant and antiproliferative activities of mango (*Mangifera indica* L.) flesh and peel. Food Chem.

[13] Tsai PJ, Tsai TH, Yu CH, Ho SC (2007). Comparison of NO-scavenging and NO-suppressing activities of different herbal teas with those of green tea. Food Chem.

[14] Wang XG, Shao F, Wang HJ, Yang L, Yu JF, Gong DQ, Gu ZL (2013). MicroRNA-126 expression is decreased in cultured primary chicken hepatocytes and targets the sprouty-related EVH1 domain containing 1 mRNA. Poultry Sci.

[15] Tang S, Lv Y, Chen H, Adam A, Cheng Y, Hartung J, Bao E (2014). Comparative analysis of αβ-crystallin expression in heat-stressed myocardial cells *in vivo* and *in vitro*. PloS one.

[16] Xia D, Fan Y, Zhang P, Fu Y, Ju M, Zhang X (2013). Protective effects of the flavonoid-rich fraction from rhizomes of *Smilax glabra* Roxb. on carbon tetrachloride-induced hepatotoxicity in Rats. J Membrane Biol.

[17] Pareek A, Godavarthi A, Issarani R, Nagori BP (2013). Antioxidant and hepatoprotective activity of *Fagonia schweinfurthii* (Hadidi) Hadidi extract in carbon tetrachloride induced hepatotoxicity in HepG2 cell line and rats. J Ethnopharmacol.

[18] Oskoueian E, Abdullah N, Ahmad S (2012). Phorbol esters isolated from Jatropha meal induced apoptosis-mediated inhibition in proliferation of Chang and Vero cell lines. IJMS.

[19] Vandesompele J, De Preter K, Pattyn F, Poppe B, Van Roy N, De Paepe A, Speleman F (2002). Accurate normalization of real-time quantitative RT-PCR data by geometric averaging of multiple internal control genes. Genome Biol.

[20] Lu Y, Sarson AJ, Gong J, Zhou H, Zhu W, Kang Z, Yu H, Sharif S, Han Y (2009). Expression profiles of genes in toll-like receptor-mediated signaling of broilers infected with *Clostridium perfringens*. Clin Vaccine Immunol.

[21] Leshchinsky TV, Klasing KC (2001). Divergence of the inflammatory response in two types of chickens. Dev Comp Immunol.

[22] Ojano-Dirain C, Toyomizu M, Wing T, Cooper M, Bottje WG (2007). Gene expression in breast muscle and duodenum from low and high feed efficient broilers. Poultry Sci.

[23] Oskoueian E, Abdullah N, Ahmad S (2012). Phorbol esters from Jatropha meal triggered apoptosis, activated PKC-δ, caspase-3 proteins and down-regulated the proto-oncogenes in MCF-7 and HeLa cancer cell lines. Molecules.

[24] Hossain M, Rahman A (2011). Chemical composition of bioactive compounds by GC-MS screening and anti-fungal properties of the crude extracts of cabbage samples. Asian J Biotechnol.

[25] SAS (2002). Statistical Analysis System.

[26] Zhang A, Sun H, Wang X (2013). Recent advances in natural products from plants for treatment of liver diseases. Eur J Med Chem.

[27] Pasias I, Farmaki E, Τhomaidis N, Piperaki E (2010). Elemental content and total antioxidant activity of *Salvia fruticosa*. Food Anal Methods.

[28] Sathaye S, Bagul Y, Gupta S, Kaur H, Redkar R (2011). Hepatoprotective effects of aqueous leaf extract and crude isolates of *Murraya koenigii* against *in vitro* ethanol-induced hepatotoxicity model. Exp Toxicol Pathol.

[29] Deng JS, Chang YC, Wen CL, Liao JC, Hou WC, Amagaya S, Huang SS, Huang GJ (2012). Hepatoprotective effect of the ethanol extract of *Vitis thunbergii* on carbon tetrachloride-induced acute hepatotoxicity in rats through anti-oxidative activities. J Ethnopharmacol.

[30] Ng KL, Ayob MK, Said M, Osman MA, Ismail A (2013). Optimization of enzymatic hydrolysis of palm kernel cake protein (PKCP) for producing hydrolysates with antiradical capacity. Ind Crop Prod.

[31] Vijayarathna S, Sasidharan S (2012). Cytotoxicity of methanol extracts of *Elaeis guineensis* on MCF-7 and Vero cell lines. Asian Pac J Trop Biomed.

[32] Ramnath V, Rekha P, Sujatha K (2008). Amelioration of heat stress induced disturbances of antioxidant defense system in chicken by Brahma Rasayana. Evid-Based Compl Alt.

[33] Das A (2011). Heat stress-induced hepatotoxicity and its prevention by resveratrol in rats. Toxicol Mech Method.

[34] Domitrovic R, Jakovac H, Blagojevic G (2011). Hepatoprotective activity of berberine is mediated by inhibition of TNF-α, COX-2, and iNOS expression in CCl_4_-intoxicated mice. Toxicology.

[35] Yan J, Bao E, Yu J (2009). Heat shock protein 60 expression in heart, liver and kidney of broilers exposed to high temperature. Res Vet Sci.

[36] Guo S, Wharton W, Moseley P, Shi H (2007). Heat shock protein 70 regulates cellular redox status by modulating glutathione-related enzyme activities. Cell Stress Chaperones.

[37] Meng X, Harken AH (2002). The interaction between HSP70 and TNF-α expression: a novel mechanism for protection of the myocardium against post-injury depression. Shock.

[38] Guzhova IV, Darieva ZA, Melo AR, Margulis BA (1997). Major stress protein Hsp70 interacts with NF-kB regulatory complex in human T-lymphoma cells. Cell Stress Chaperones.

[39] Zhang L, Liu Q, Yuan X, Wang T, Luo S, Lei H, Xia Y (2013). Requirement of heat shock protein 70 for inducible nitric oxide synthase induction. Cell Signal.

[40] Ogbuagu M (2008). The change in physico-chemical properties of blended oils of palm origin with soyabean oil. Global J Pure Appl Sci.

[41] Makni M, Fetoui H, Gargouri N, Garoui EM, Jaber H, Makni J, Boudawara T, Zeghal N (2008). Hypolipidemic and hepatoprotective effects of flax and pumpkin seed mixture rich in ω-3 and ω-6 fatty acids in hypercholesterolemic rats. Food Chem Toxicol.

[42] Maheswari MU, Rao P (2005). Antihepatotoxic effect of grape seed oil in rat. Indian J Pharmacol.

[CR43] The pre-publication history for this paper can be accessed here:http://www.biomedcentral.com/1472-6882/14/368/prepub

